# Fibrogenesis in Human Mucosa and Muscularis Precision-Cut Intestinal Slices

**DOI:** 10.3390/cells13131084

**Published:** 2024-06-22

**Authors:** Carin Biel, Anniek Kastermans, Janneke Heidema, Martin Pehrsson, Charlotte Henstra, Joachim Mortensen, Klaas Nico Faber, Peter Olinga

**Affiliations:** 1 Department of Pharmaceutical Technology and Biopharmacy, Groningen Research Institute of Pharmacy, University of Groningen, 9713 AV Groningen, The Netherlands; c.biel@rug.nl (C.B.); c.henstra@rug.nl (C.H.); 2 Biomarkers and Research, Nordic Bioscience, 2730 Herlev, Denmark; mpe@nordicbio.com (M.P.); jhm@nordicbio.com (J.M.); 3 Department of Gastroenterology and Hepatology, University Medical Centre Groningen, 9713 GZ Groningen, The Netherlands; k.n.faber@umcg.nl

**Keywords:** inflammatory bowel disease, matrix metalloproteinases, human precision-cut intestinal slices

## Abstract

In Crohn’s Disease (CD), intestinal fibrosis is a prevalent yet unresolved complication arising from chronic and transmural inflammation. The histological assessment of CD intestines shows changes in tissue morphology in all the layers, including the mucosa and muscularis. This study aimed to determine the differences in fibrogenesis between mucosa and muscularis. Human precision-cut intestinal slices (hPCIS) were prepared from human intestine mucosa and muscularis and treated with TGF-β1 and/or PDGF-BB for 72 h. Gene and protein expression and matrix metalloproteinase (MMP) activity were determined. The basal gene expression of various fibrosis markers was higher in muscularis compared to mucosa hPCIS. During incubation, Pro-Collagen-1A1 secretion increased in muscularis but not in mucosa hPCIS. MMP gene expression increased during incubation in mucosa and muscularis hPCIS, except for MMP9, MMP12, and MMP13 in muscularis hPCIS. Incubation with TGF-β1 caused increased COL1A1 expression in the mucosa but not in muscularis hPCIS. In muscularis hPCIS, TGF-β1 treatment caused a decrease in MMP1 and CTSK expression, while MMP13 was increased. In the presence of TGF-β1, protease inhibitor expression was stable, except for SERPINE1, which was increased in muscularis hPCIS. We conclude that fibrogenesis is more pronounced in muscularis hPCIS compared to mucosa hPCIS, especially when stimulated with TGF-β1.

## 1. Introduction

Intestinal fibrosis (IF) is a common complication of patients suffering from inflammatory bowel diseases (IBDs), especially Crohn’s Disease (CD) [1,2]. IF is characterized by an increased deposition of extracellular matrix (ECM) and smooth muscle cell hypertrophy [3,4,5]. This hypertrophy causes thickening of the intestinal wall and narrowing of the intestinal lumen, ultimately leading to obstruction. Currently, the only treatment option is the resection of the affected part of the intestine. Unfortunately, IF is recurrent in approximately one-third of CD patients [2].

It is thought that IF is caused by chronic inflammation and an excessive wound-healing response. During fibrogenesis, intestinal fibroblasts are activated and differentiate into myofibroblasts. These myofibroblasts produce ECMs and contract in an attempt to heal the injured part of the intestine [1,6]. When the wound is closed, myofibroblasts undergo apoptosis, and the wound-healing response ends. In the case of CD, as there is chronic inflammation, the continuous wound-healing response eventually leads to fibrosis [7,8].

Intestinal inflammation is transmural in CD patients; tissue changes are detected from the mucosa until the serosa, including the muscularis propria [4,5]. Moreover, the histological assessment of IF biopsies has shown that not only do the mucosa and submucosa show signs of fibrosis but the muscularis and serosa also have alterations in their normal morphology [4,5]. For example, collagen bundles are present in the muscularis layer of IF patients, smooth muscle cells (SMCs) of the muscularis are hypertrophic, and SMC bundles are found outside the muscularis [3,5]. Thus, the mucosa as well as the muscularis are involved in the fibrogenesis of CD patients.

In vitro, it is difficult to study the differences in distinct tissue layers, like the mucosa and the muscularis. First, this is caused by the lack of extracellular matrix and cell–cell interactions in in vitro cell culture systems. It is well known that the response of cells, for example, fibroblasts or SMCs, depends on their extracellular environment [9,10,11], which is different in the mucosa and muscularis. In vivo animal studies may resolve this problem. However, there is a translational gap between animals and humans, also in fibrosis-related molecular pathways. In addition, the use of in vivo models does not reduce the use of laboratory animals as intended by the 3R principle (reduction, refinement and replacement) [12,13]. Human precision-cut intestinal slices (hPCIS) do overcome the problems described above. Since hPCIS consist of intact human intestinal tissue layers, ECM and cell–cell interactions remain present during the culture of hPCIS. Therefore, we hypothesize that the cells present in the hPCIS respond in a more representative way to stimuli compared to cells in traditional cell culture. Since we are able to produce and use PCISs derived from human tissue, we omit the use of laboratory animals and strengthen the translational potential of the PCIS model.

It is unclear how fibrosis in mucosa and muscularis develops. However, it is well-established that the transforming growth factor (TGF)-β1 and platelet-derived growth factor (PDGF)-BB plays a major role in fibrosis in the intestine as well as in other organs [3,14,15,16]. The aim of this study was to determine the differences in fibrogenesis between the mucosa and the muscularis. For this purpose, we obtained (healthy) human intestines, prepared hPCIS from both the mucosa and muscularis, and treated these slices with TGF-β1 and/or PDGF-BB. Subsequently, we analyzed the differences in the fibrotic response by the quantitative reverse transcriptase polymerase chain reaction (RT-qPCR) analysis of genes encoding for proteins involved in fibrogenesis, determined the production of ECM proteins by hPCIS, and analyzed the activity of matrix metalloproteinases (MMPs) by measuring ECM breakdown products in the culture medium. Lastly, we determined ECM deposition by calculating the collagen formation/degradation ratio and Sirius Red staining.

## 2. Materials and Methods

### 2.1. Human Intestine

Healthy intestinal tissue for research was obtained in accordance with Dutch legislation and the Code of Conduct for dealing responsibly with human tissue in the context of health research (www.federa.org), refraining from the need for written consent for “further use” of coded-anonymous human tissue. The tissue originated from the resected intestine (i.e., proximal jejunum) of patients who underwent a pylorus-preserving pancreaticoduodenectomy (PPPD) (Table 1). After resection, the intestine was immediately stored in ice-cold Krebs-Henseleit buffer (KHB) and processed immediately after collection.

### 2.2. Human Intestinal Mucosa and Muscularis Slices

**Figure 1:**
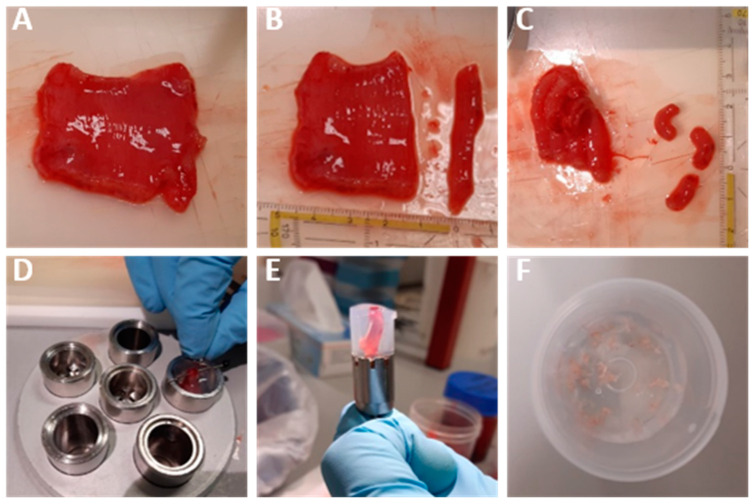
Preparation of muscularis hPCIS: (**A**) Mucosa and muscularis were separated by cutting through the submucosa. (**B**) The muscularis layer was divided into 0.5 cm sheets, and (**C**) the sheets were cut into approximately 1.5 cm pieces. (**D**) The muscularis cores were prepared by fixating the muscularis pieces at the top and bottom of the embedding unit by using metal pins, and subsequent low-melting agarose was used to fixate the tissue. (**E**) The muscularis core should have a straight orientation in the solidified agarose; muscularis hPCIS were prepared using the Krumdieck tissue slicer using the lowest arm speed, fastest blade speed, and blade replacement for every other core. (**F**) After slicing, the agarose was removed, and the slices were stored in an ice-cold KREBS buffer until incubation.

To prepare hPCIS from mucosal tissue, tissue processing and slicing procedures were performed as described before [17]. Human muscularis propria (Figure 1A) has not been used to prepare slices before. Similar to the preparation of mucosal slices, the muscularis was cut into sheets of approximately 0.5 cm thick and divided into pieces of approximately 1 cm long (Figure 1B,C). Subsequently, muscularis cores were prepared using pins to keep the muscularis in place, and a cold embedding unit was used to promote the solidification of the low-melting agarose (Figure 1D,E). Slices of mucosa and muscularis hPCIS were prepared in ice-cold KHB by using a Krumdieck tissue slicer (Alabama Research and Development, Munford, AL, USA). Muscularis and muscularis hPCIS weighed around 3 mg and 10 mg, respectively, and were stored in ice-cold KHB until the start of the experiment (Figure 1F).

### 2.3. Incubation of Mucosa and Muscularis Slices

Mucosa as well as muscularis hPCIS were incubated separately in 1.3 mL of culture medium (Advanced DMEM/F12 (ADF) (Gibco™, Life Technologies, Bleiswijk, The Netherlands, Cat. No. 12634010) supplemented with 2 mM Glutamax (Gibco™, Life Technologies, Bleiswijk, The Netherlands, Cat. No. 35050061), 50 μg/mL Gentamycin (Gibco™, Life Technologies, Bleiswijk, The Netherlands, Cat. No. 15750037), and 2.5 μg/mL Amphotericin B (Gibco™, Life Technologies, Bleiswijk, The Netherlands, Cat. No. 15290026) in 12-well plates. ADF was chosen as the standard culture medium for both mucosa and muscularis slices because pilot experiments showed a slightly better viability of slices cultured in ADF compared to slices incubated in Williams medium E (WME), DMEM, or the DMEM/F12 medium (Supplementary Figure S1). To stimulate fibrogenesis, the slices were exposed to 5 ng/mL TGF-β1 (Peprotech, Cranbury, NJ, USA, Cat. No. 100-21) and/or 50 ng/mL PDGF-BB (Peprotech, Cranbury, NJ, USA, Cat. No. 100-14B) for 72 h. TGF-β1 and PDGF-BB were stored and dissolved according to the manufacturer’s instructions and added to the culture medium immediately before incubation. The slices were incubated for 72 h at 37 °C, 80% O_2_, and 5% CO_2_ in a shaking incubator; the culture medium was collected and replaced every 24 h.

### 2.4. ATP/Protein Determination

Immediately after slicing and after incubation, the mucosa and muscularis slices were collected individually in 0.5 mL or 1 mL sonication solution (70% alcohol and 2 mM EDTA), respectively, snap-frozen, and stored at −80 °C until measurement (*n* = 3 per condition). ATP content and ATP/protein ratios were determined as described previously [18].

### 2.5. Histochemical and Immunohistochemical Staining

The mucosa and muscularis slices were fixed for 24 h in 4% buffered formalin, processed, and embedded in paraffin according to standard procedure. Subsequently, 4 μm sections were prepared and stained to evaluate the general morphology (hematoxylin and eosin staining, i.e., HE), collagen/ECM fiber morphology (Sirius Red staining), and activation of fibroblasts to myofibroblasts (α-SMA immunohistochemistry staining).

HE staining and Sirius Red staining (Picro Sirius Red Staining kit, Abcam, Cambridge, MA, USA, Cat. No. ab150681) were performed according to standard procedure and immunohistochemistry, as previously described [18]. For α-smooth muscle actin (SMA) staining, the sections were incubated with primary antibody (Cell signalling, Technology PA, Leiden, The Netherlands, Cat. No. 19245, 1:1000) and with the secondary antibody (goat-anti-rabbit HRP, Dako, Glostrup, Denmark, Cat.No. P0448, 1:50).

### 2.6. mRNA Expression Analysis

Immediately after slicing and after incubation, pooled slices (6 per condition) were snap-frozen and stored at −80 °C until RNA isolation. RNA isolation, cDNA synthesis, and RT-qPCR were performed as previously described [18]. mRNA expression was calculated using the 2-ΔCt method, with *YWHAZ* as a reference gene. For the generation of heatmaps, Z-values were calculated using the 2-ΔCt-average and 2-ΔCt-standard deviation from individual experiments. Heatmaps were generated using the Morphous online software (https://software.broadinstitute.org/morpheus/ (accessed on 18 June 2024)). Hierarchical clustering analysis was performed by the average linkage clustering method using Pearson correlation.

### 2.7. Pro-Collagen I Alpha I ELISA

The culture medium was collected every 24 h, centrifuged to remove debris, and stored at −80 °C until measurement. The Human Pro-Collagen I alpha I SimpleStep enzyme-linked immunosorbent assay (ELISA)^®^ kit (Abcam, Cambridge, MA, USA, Cat. No. ab210966) was used according to the manufacturer’s instructions. Mucosa medium samples were diluted 15 times, while muscularis slices were diluted 20–200 times. OD (450 nm) was measured with a BioTek Synergy HT (BioTek Instruments, Winooski, VT, USA).

### 2.8. Biomarker Assays

Type I and type III collagen remodeling were assessed by quantifying the C1M [19], C3M [20], PRO-C1 [21], and PRO-C3 [22] biomarkers in the collected hPCIS culture medium using competitive ELISA. C1M and C3M quantify fragments of the MMP-2, -9, and -13 catalyzed degradations of type I collagen and the MMP-9 catalyzed degradation of type III collagen, respectively, whereas PRO-C1 and PRO-C3 quantify N-terminal pro-peptides, reflecting type I and type III collagen formation. ELISA manufacturing and biomarker measurements were conducted at Nordic Bioscience A/S (Herlev, Denmark) according to the previously described protocols [19,20,21,22].

### 2.9. Statistical Analysis

GraphPad Prism (version 9.0, GraphPad Software Inc., Boston, MA, USA) was used to perform the statistical analysis. The data were considered paired and non-parametric. For the comparison of values at the start of incubation and after incubation, or to compare different treatments, the Friedman test with Dunn’s post-hoc test was used (using Δ Ct for gene expression data). A *p*-value of <0.05 was considered statistically significant.

## 3. Results

### 3.1. Human Intestinal Mucosa and Muscularis Slices

**Figure 2:**
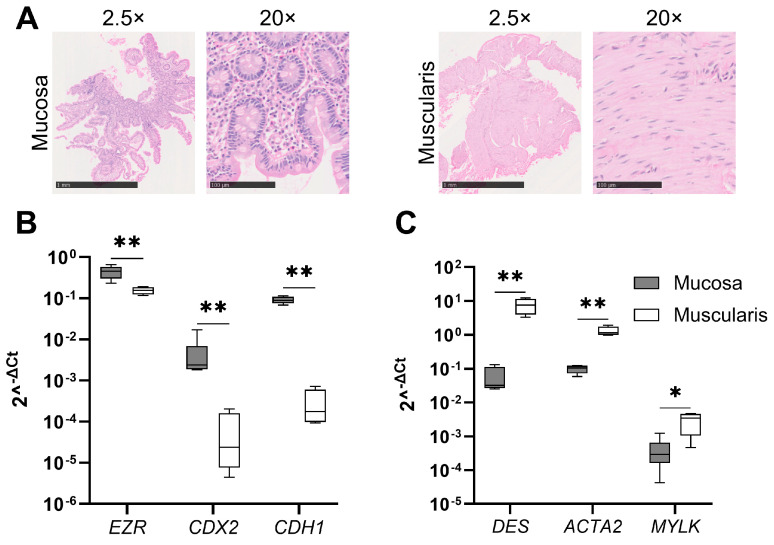
Confirmation of separated mucosa and muscularis hPCIS. (**A**) Representative images of HE-stained mucosa and muscularis hPCIS immediately after slicing. The bars indicate 1 mm (2.5×) magnification and 100 μm (20×) magnification. (**B**) Gene expression of mucosa/epithelial markers in the mucosa and muscularis hPCIS. (**C**) Gene expression of muscle markers in the mucosa and muscularis hPCIS. The bars indicate minimum, maximum, and median values. * *p* < 0.05 and ** *p* < 0.01.

Human muscularis propria has not been used to prepare slices before. We developed and optimized the procedure of preparing human muscularis PCISs (Figure 1), which was comparable to the preparation of human mucosa PCISs as described previously [17]. We confirmed tissue specificity using HE staining and quantitative reverse transcriptase polymerase chain reaction (RT-qPCR) analysis (Figure 2A–C). Mucosa and muscularis hPCIS fixated immediately after slicing clearly represented the mucosal and muscularis layers of the intestine. HE staining revealed the typical morphology of the mucosa, including the villi, crypts, muscularis mucosae, and lamina propria in mucosa hPCIS (Figure 2A). The HE staining of muscularis hPCIS clearly showed the typical smooth muscle layers of the muscularis propria (Figure 2A). In some muscularis hPCIS, both the longitudinal and the circular muscle layers were visualized using HE staining. In both mucosa and muscularis hPCIS, the submucosal layer was present, as shown by the presence of irregular connective tissue and blood vessels. The gene expression level of epithelial markers (*EZR*, *CDX2*, and *CDH1*) was 10–1000 times higher in mucosa hPCIS compared to muscularis hPCIS (Figure 2B), while muscle markers (*DES*, *ACTA2*, and *MYLK*) were higher in the muscularis hPCIS compared to the mucosa hPCIS (Figure 2C). Altogether, these results confirm that the mucosa and muscularis were successfully dissected, and the slices showed a clear tissue-specific histology and gene expression pattern at the start of the ex vivo incubations.

### 3.2. hPCIS Viability

To select the best culture medium to preserve tissue viability, mucosa and muscularis hPCIS were incubated in a WME, DMEM, DMEM-F12, or Advanced DMEM-F12 medium for up to 72 h. The ATP/protein ratios of mucosa hPCIS were similar between mucosa hPCIS cultures in the four different media, except for mucosa hPCIS incubated for 24 h in DMEM-F12; these slices had a significantly lower ATP/protein ratio, indicating lower viability (Supplementary Figure S1A). The scoring of the HE staining (Supplementary Figure S1B) of mucosa hPCIS did not show significant differences between slices cultured in the different media (Supplementary Figure S1C). ATP/protein ratios of muscularis hPCIS cultured in the different media showed a significantly higher ATP/protein ratio of muscularis hPCIS incubated for 48 h in Advanced DMEM-F12 (Supplementary Figure S1D). The morphology of HE-stained muscularis hPCIS appeared very similar (Supplementary Figure S1E). Since Advanced DMEM-F12 appeared to be the best culture medium for the mucosa hPCIS as well (lowest Park score at all time points and highest ATP/protein ratio at two timepoints), the Advanced DMEM-F12 medium was selected as the most optimal culture medium for further experiments.

### 3.3. Fibrogenesis during the Incubation of hPCIS Is more Pronounced in Muscularis Compared to Mucosa

**Figure 3:**
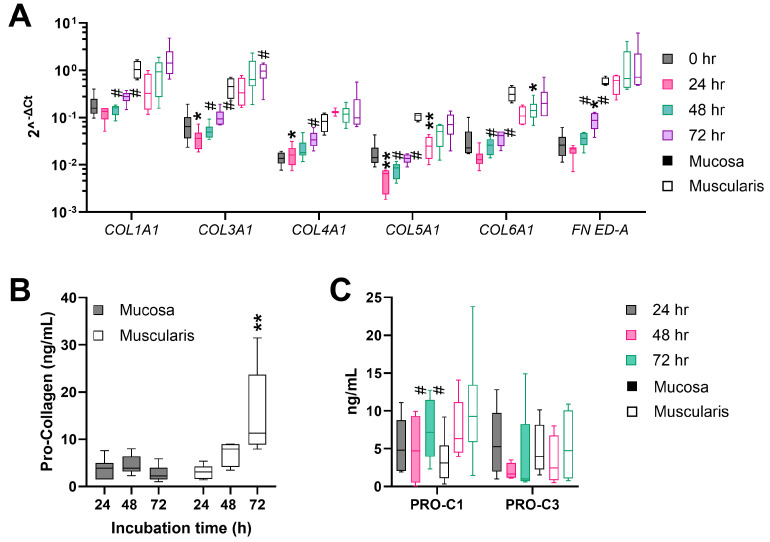
ECM production in the mucosa and muscularis hPCIS during incubation for 72 h. Mucosa and muscularis hPCIS were incubated for 24–72 h in A-DMEM-F12. The culture medium was collected every 24 h. (**A**) Gene expression analysis of ECM-related genes. * *p* < 0.05, ** *p* < 0.01 compared to 0 h, and # *p* < 0.05, ## *p* < 0.01 compared to 24 h. (**B**) The Pro-collagen 1A1 N-peptide (PINP) content in the culture medium was determined using the ab210966 ELISA kit. ** *p* < 0.01 compared to 24 h. (**C**) PINP and PIIINP content in the culture medium was determined using competitive ELISAs. ## *p* < 0.01 compared to 48 h. The bars indicate minimum, maximum, and median values. The closed bars indicate values obtained from mucosa hPCIS, and the open bars show muscularis hPCIS.

To determine whether fibrogenesis is induced upon hPCIS culture, hPCIS were incubated for 72 h and gene expression analysis and pro-collagen secretion were determined. The basal gene expression of genes encoding for ECM proteins and inhibitors of MMPs (*COL1A1*, *COL3A1*, *COL4A1*, *COL5A1*, *COL6A1*, *FN ED-A*, *CTSK*, *MMP-2*, *TIMP-1*, *TIMP-2*, and *SERPINE1*) was significantly higher (10–100 fold) in muscularis hPCIS compared to mucosa hPCIS immediately after slicing (Supplementary Figure S2), indicating a higher basal ECM production and turnover in the muscularis hPCIS. The gene expression of ECM proteins was downregulated in the first 24 h of incubation but recovered and increased after 48- and 72-hour incubation in mucosa as well as in muscularis hPCIS (Figure 3A). This was more pronounced in the mucosa compared to the muscularis. Mucosal messenger RNA levels of *COL1A1*, *COL3A1*, *COL4A1*, *COL5A1*, *COL6A1*, and *FN ED-A* were significantly enhanced after 72 h of incubation compared to 24 h of incubation.

As a measure of collagen formation, the levels of pro-collagen 1A1 N-terminal pro-peptide in the culture medium were determined. Mucosal hPCIS secreted 3.9 ng/mL (median) pro-collagen 1A1 during the first 24 h of incubation, a production rate that remained stable during the following 24–72 h of incubation (Figure 3B). In muscularis hPCIS, the pro-collagen 1A1 secretion was similar (median 3.1 ng/mL) to mucosa hPCIS in the first 24 h of incubation but increased significantly over time (median of 11.3 ng/mL between 48 and 72 h, Figure 3B). Using ELISAs, the formation rates of collagen 1 and 3 (PRO-C1 and PRO-C3) were determined. PRO-C1 production levels increased slightly in the culture medium collected after 72 h (median of 7.2 ng/mL/24 h) of mucosa hPCIS incubation compared to the culture medium collected after 48 h of incubation (median of 4.7 ng/mL/24 h), while PRO-C3 decreased over incubation time (median values of 5.3, 1.7, and 1.0 ng/mL/24 h, ns) (Figure 3C). PRO-C1 in the culture medium from muscularis hPCIS collected after 48 and 72 h (median values of 6.3 and 9.3 ng/mL/24 h, respectively) seemed to be higher than those collected after 24 h (median of 3.1 ng/mL/24 h). However, this was not significant after 72 h of incubation. PRO-C3 production levels from muscularis hPCIS remained stable over the incubation time (median values of 4.0, 2.4, and 4.7 ng/mL/24 h). Altogether, the mRNA expression of collagens (Figure 3A) is reflected in the collagen formation rates (Figure 3B,C).

When mucosa and muscularis hPCIS were compared both at the gene and protein expression levels, the ECM molecules had a higher expression level in muscularis hPCIS compared to mucosa hPCIS, especially after 72 h of culture. Of note, we checked whether (myo)fibroblasts were present in mucosa hPCIS during culture. Gene expression analysis revealed that (myo)fibroblast markers were decreased upon incubation (especially *ACTA2*, Supplementary Figure S3). However, using immunohistochemistry, we showed that αSMA+ (myo)fibroblasts were preserved during the 72-hour incubation time (Supplementary Figure S3). This indicated that ECM-producing cells were still present in the mucosa hPCIS up to 72 h of incubation.

**Figure 4:**
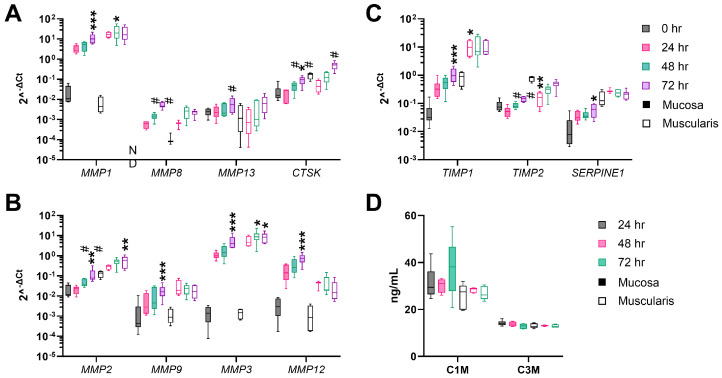
ECM degradation in mucosa and muscularis hPCIS during incubation for 72 h. Mucosa and muscularis hPCIS were incubated for 24–72 h in A-DMEM-F12. Gene expression analysis of ECM remodeling-related genes was performed. The gene expression of (**A**) collagenases; (**B**) gelatinases, stromelysin, and macrophage elastase; (**C**) inhibitors of MMPs; and (**D**) C1M (MMP-2, -9, and -13 degradation products of type I collagen) and C3M (MMP-9 degradation product of type III collagen) were determined in the culture medium using competitive ELISAs. The bars indicate minimum, maximum, and median values. The closed bars indicate values obtained from mucosa hPCIS, and the open bars show muscularis hPCIS. * *p* < 0.05, ** *p* < 0.01, and *** *p* < 0.005 compared to 0 h; # *p* < 0.05 and ## *p* < 0.01 compared to 24 h.

Fibrogenesis is counteracted by the remodeling of the ECM by proteolytic enzymes, such as MMPs and Cathepsin K (*CTSK*), and inhibitors of these enzymes, such as tissue inhibitors of MMPs (TIMPs) and serine proteinase inhibitor E1 (*SERPINE1*). The mucosal gene expression of all MMPs (*MMP-1*, *-2*, -*3*, *-8*, *-9*, *-12*, and -*13*) and *CTSK* was upregulated upon culture (Figure 4A,B). In the muscularis, *MMP-1*, *-8*, *-9*, *-12*, and *-13* were not significantly upregulated, while *CTSK*, and *MMP-2* and *-3* were significantly upregulated after 72 h of incubation compared to muscularis hPCIS immediately after slicing (Figure 4A,B). Inhibitors of MMPs (*TIMP-1* and *-2*, and *SERPINE1*) were significantly increased upon culture in mucosa hPCIS but not in muscularis hPCIS compared to hPCIS immediately after slicing (Figure 4C). Using biomarker assays, the degradation products of MMP-2, -9, and -13, e.g., degraded collagen 1 (C1M) and MMP-9-degraded collagen 3 (C3M), were determined in the culture medium. The ECM degradation rate, represented by C1M and C3M concentrations, remained stable in the culture medium of mucosa and muscularis hPCIS upon culture (Figure 4D). The MMP expression increased upon hPCIS incubation, indicating that an inflammatory or wound-healing response is initiated upon culture. However, the concentration of ECM degradation products remained stable, indicating that the transcribed genes were not translated to active enzymes or that these enzymes were inhibited by their inhibitors. The latter was supported by the increased mRNA expression of MMP inhibitors upon culture.

**Figure 5:**
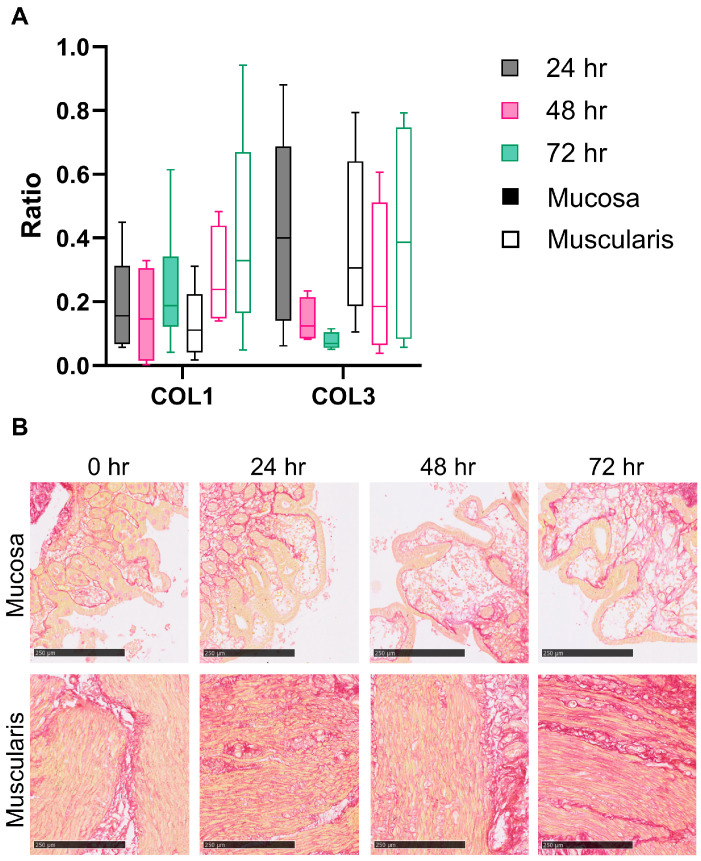
Fibrogenesis in mucosa and muscularis hPCIS during incubation for 72 h. Mucosa and muscularis hPCIS were incubated for 24–72 h in A-DMEM-F12. The culture medium was collected every 24 h. (**A**) Type I and III collagen formation and degradation products were determined, and PRO-C1/C1M and PROC3/C3M ratios were calculated as indicators of net ECM production. The bars indicate minimum, maximum, and median values. The closed bars indicate values obtained from mucosa hPCIS, and the open bars show muscularis hPCIS. (**B**) Representative images of Sirius Red-stained mucosa and muscularis hPCIS. The bars indicate 250 μm (10×) magnification. Red indicates collagen bundles, and yellow indicates the cytoplasm.

To assess whether the changes in collagen production and degradation result in altered collagen deposition, we calculated collagen formation/degradation product ratios as an indicator of net collagen formation or degradation. Moreover, Sirius Red staining was used to visualize collagens in the hPCIS. We found that the PRO-C1/C1M ratio is stable in mucosa hPCIS, while it steadily increases upon the incubation of muscularis hPCIS, although this was not significant after 72 h of incubation (Figure 5A). The PRO-C3/C3M ratio tended to decrease upon the incubation of mucosa hPCIS, while the PRO-C3/C3M ratio remained stable in the culture medium from muscularis hPCIS (Figure 5A). Visually, the thickness of collagen fibers was increased upon the incubation of muscularis hPCIS but not of mucosa hPCIS, indicating increased collagen deposition in the muscularis hPCIS (Figure 5B). In summary, genes encoding for proteins involved in fibrosis are differently expressed between mucosa and muscularis upon ex vivo culture (Supplementary Figures S4 and S5), resulting in a more pronounced fibrotic response in the muscularis compared to the mucosa.

### 3.4. TGF-β1 Stimulates Fibrogenesis in Muscularis hPCIS but Not in Mucosal hPCIS

To determine whether the fibrotic response could be (further) enhanced, we exposed mucosa and muscularis hPCIS to TGF-β1 and/or PDGF-BB for 72 h. To assess whether the mucosa and muscularis remained viable during incubation in the presence of TGF-β1 and/or PDGF-BB, ATP/protein ratios were determined, and the histology was assessed using HE staining. After the slicing procedure, the mean ATP/protein ratio was 1.9 pmol/μg for mucosa hPCIS and 3.4 pmol/μg for muscularis hPCIS, indicating that the slices were viable immediately after the slicing procedure. After 24 h, PDGF-BB treatment resulted in a significantly lower ATP/protein ratio in both mucosa and muscularis hPCIS compared to non-treated hPCIS (Supplementary Figure S6A,B). After 48 h of TGF-β1 and PDGF-BB treatment, the ATP/protein ratio in muscularis hPCIS was higher compared to controls (Supplementary Figure S6B). None of the treatments (TGF-β1 and/or PDGF-BB) caused a significant increase or decrease in ATP/protein ratio in the mucosa or muscularis slices after 72 h of incubation compared to non-treated hPCIS (Supplementary Figure S6A,B). HE staining did not reveal differences in the histology of both mucosa and muscularis hPCIS up to 72 h treatment with or without TGF-β1 and/or PDGF-BB (Supplementary Figure S6C,D). These results show that the viability of both types of hPCIS is not significantly affected by exposure to 5 ng/mL TGF-β and/or 50 ng/mL PDGF-BB for 72 h.

**Figure 6:**
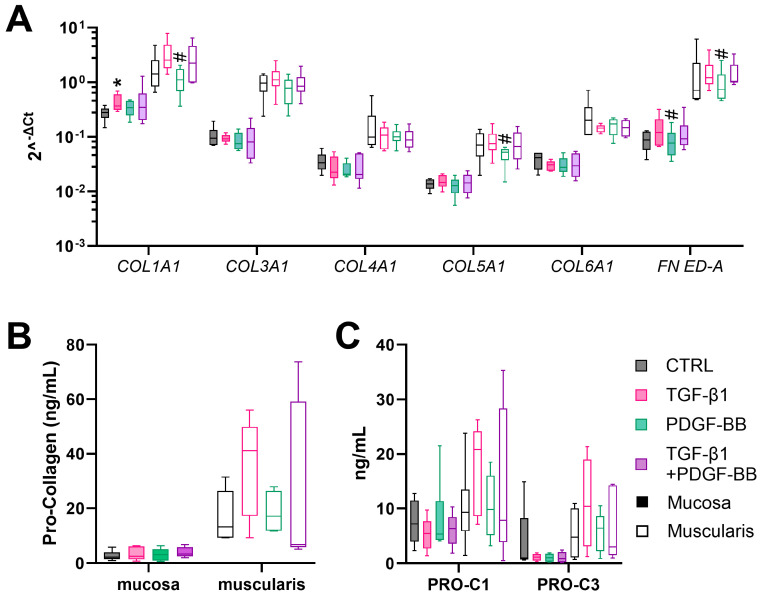
ECM production in the mucosa and muscularis hPCIS upon TGF-β1 and/or PDGF-BB treatment. Mucosa and muscularis hPCIS were incubated for 72 h in A-DMEM-F12 (CTRL) with and without 5 ng/mL TGF-β1 and/or 50 ng/mL PDGF-BB. (**A**) Gene expression analysis of ECM-related genes. (**B**) Pro-collagen 1A1 N-peptide (PINP) content in the culture medium was determined using the ab210966 ELISA kit. (**C**) PINP and PIIINP content in the culture medium was determined using competitive ELISAs. The bars indicate minimum, maximum, and median values. The closed bars indicate values obtained from mucosa hPCIS, and the open bars show muscularis hPCIS. * *p* < 0.05 compared to CTRL and # *p* < 0.01 compared to +TGF-β1.

TGF-β1 and/or PDGF-BB treatment for 72 h had no clear effect on ECM production in mucosal hPCIS (Figure 6). Only *COL1A1* mRNA levels were significantly increased in mucosa hPCIS (Figure 6A). However, this was not reflected in Pro-COL1A1 N-peptide (Figure 6B), PRO-C1, or PRO-C3 levels (Figure 6C). In contrast, muscularis hPCIS did show a trend towards a pro-fibrotic response upon TGF-β1 treatment. The expression of genes encoding ECM proteins (*COL1A1*, *COL3A1*, *COL5A1*, and *FN ED-A*), pro-COL1A1 pro-peptide secretion, and collagen 1 and 3 formation products (PRO-C1 and PRO-C3) were increased in four to six of the six experiments, but this was not significant (Figure 6C).

PDGF-BB did not enhance fibrogenesis upon PCIS culture or in combination with TGF-β1 (Figure 5). In addition, TGF-β1 or PDGF-BB did not stimulate myofibroblast differentiation or proliferation, as shown by the gene expression of fibroblast markers and protein expression of α-SMA (Supplementary Figures S3, S7, and S8).

**Figure 7:**
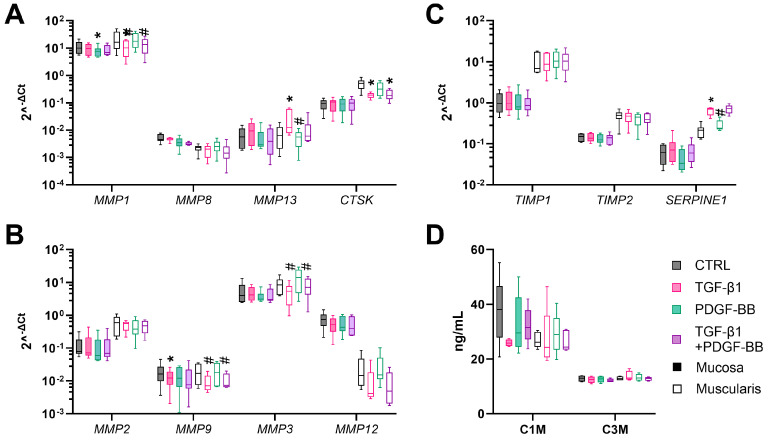
ECM degradation in mucosa and muscularis hPCIS upon TGF-β1 and/or PDGF-BB treatment. Mucosa and muscularis hPCIS were incubated for 72 h in A-DMEM-F12 with and without 5 ng/mL TGF-β1 and/or 50 ng/mL PDGF-BB. Gene expression analysis of ECM remodeling-related genes was performed. The gene expression of (**A**) collagenases; (**B**) gelatinases, stromelysin, and macrophage elastase; (**C**) inhibitors of MMPs; and (**D**) C1M (MMP-2, -9, and -13 degradation products of type I collagen) and C3M (MMP-9 degradation product of type III collagen) were determined in the culture medium using competitive ELISAs. The bars indicate minimum, maximum, and median values. The closed bars indicate values obtained from mucosa hPCIS, and the open bars show muscularis hPCIS. * *p* < 0.05 compared to CTRL h, # *p* < 0.05, and ## *p* < 0.01 compared to +TGF-β1.

In muscularis hPCIS, ECM degradation was also affected by TGF-β1. This effect is shown by a significantly lower gene expression of the ECM-degrading enzymes *MMP1* and *CTSK* upon TGF-β1 treatment (Figure 6A,B). Interestingly, *MMP13*, also known as collagenase-3, had a higher expression level in muscularis hPCIS incubated with TGF-β1 (Figure 7). The levels of *TIMP-1* and -*2* were not affected by TGF-β1 treatment, but *SERPINE1* had a higher expression level in muscularis hPCIS incubated with TGF-β1 (Figure 7C). In mucosa hPCIS, the effect of TGF-β1 on MMPs and protease inhibitors was less pronounced (Figure 6A–C). To assess its effect on MMP activity, the ECM degradation products C1M and C3M were measured in the culture medium and appeared to be similar between all the treatments in both mucosa and muscularis hPCIS (Figure 7D).

**Figure 8:**
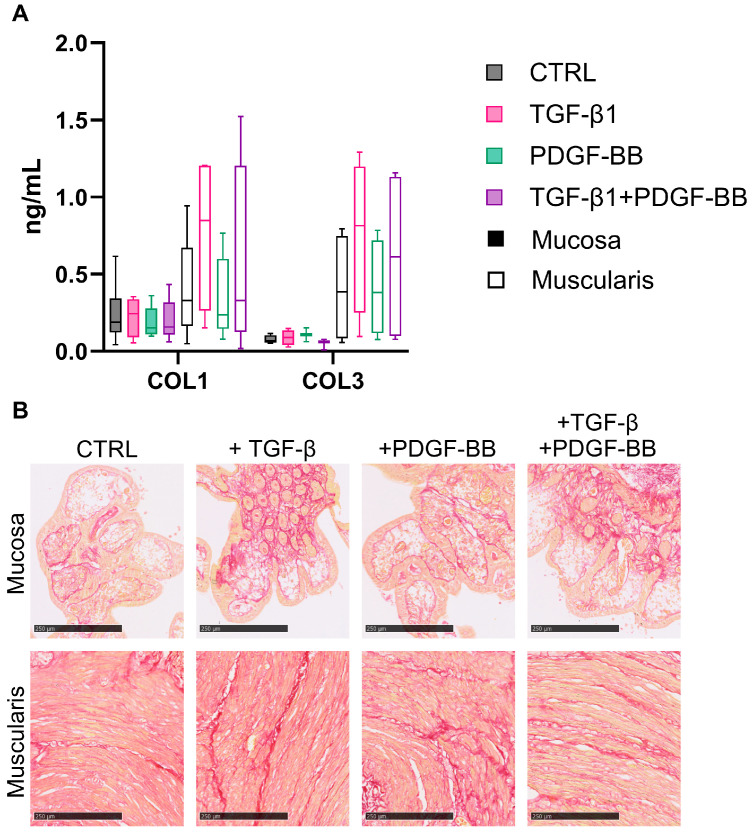
Fibrogenesis in the mucosa and muscularis hPCIS upon TGF-β1 and/or PDGF-BB treatment. Mucosa and muscularis hPCIS were incubated for 72 h in A-DMEM-F12 (CTRL) with and without 5 ng/mL TGF-β1 and/or 50 ng/mL PDGF-BB. (**A**) Type I and III collagen formation and degradation products were determined, and the formation/degradation ratio was calculated as an indicator of net ECM production. The bars indicate minimum, maximum, and median values. The closed bars indicate values obtained from mucosa hPCIS, and the open bars show muscularis hPCIS. (**B**) Representative images of Sirius Red-stained mucosa and muscularis hPCIS. The bars indicate 250 μm (10×) magnification. Red indicates collagen bundles, and yellow indicates the cytoplasm.

In addition, for TGF-β1- and/or PDGF-BB-treated hPCIS, we calculated collagen formation/degradation product ratios and performed Sirius Red staining to determine the net result of collagen formation and degradation. In mucosa hPCIS, there was no clear effect on collagen deposition by either TGF-β1 or PDGF-BB (Figure 8A). In muscularis hPCIS, the effect of TGF-β1 seemed to be more pronounced since the PRO-C1/C1M and PRO-C3/C3M ratios were higher, albeit not significant, in the culture medium from TGF-β1-treated muscularis hPCIS (Figure 8A). Sirius Red staining did not show clear effects on collagen expression (Figure 8B). If the gene expression of various fibrosis-related genes is taken into account, the pro-fibrotic effect of TGF-β1 is much clearer in muscularis hPCIS compared to mucosa hPCIS (Supplementary Figures S7 and S8). Altogether, the fibrogenesis was more pronounced in muscularis hPCIS compared to mucosa hPCIS, both upon culture and TGF-β1 treatment.

## 4. Discussion

Ex vivo cultures of PCISs have been used to study intestinal fibrosis. However, it has been shown that fibrogenesis in human PCISs (hPCIS) is not as pronounced as in mouse or rat PCISs [23]. A main difference between human and rodent PCISs is the composition of the PCIS; the hPCIS model lacks the muscularis externa since this layer is removed before the preparation of hPCIS. The aim of this study was therefore to determine the differences in fibrogenesis between mucosa and muscularis hPCIS. Thus, we prepared hPCIS from mucosa as well as from the muscularis and showed that the muscularis hPCIS provoked a more pronounced fibrogenic signature in comparison to the mucosa hPCIS.

It is clear that the muscular tissue of the intestines plays a role in the fibrogenesis in CD patients [3,5]. In this study, we demonstrated a significant role of the collagen-producing muscularis propria in fibrosis development. One of the reasons for the role of the muscularis in fibrogenesis might be the relatively high number of cells that can produce ECMs. In the mucosa, there is a thin layer of SMCs (muscularis mucosae) and different populations of fibroblasts, while the muscularis propria mainly consists of SMCs that can potentially produce ECMs. This leads to a difference in the proportion of cells that might be able to produce ECMs in the mucosa versus the muscularis [5,24]. This is reflected in the gene expression of collagens and fibronectin, which are higher in muscularis hPCIS compared to mucosa hPCIS, indicating that there is a higher proportion of cells expressing ECM molecules. Interestingly, while ECM gene expression was generally higher in muscularis hPCIS, the gene expression of ECM remodeling proteins was generally lower in the muscularis hPCIS, and the gene expression of the proteins inhibiting ECM remodeling was higher compared to the mucosa hPCIS. All these slight differences in gene expression might result in a net higher contribution to the fibrogenesis of the muscularis propria compared to the mucosa, as shown in collagen secretion and deposition. This steady increase in collagen production and deposition in the muscularis over a 72-hour culture period has been linked to a fibrogenic response. This response is induced by both warm and cold ischemic injuries, as well as by mechanical stress experienced before and during the slicing procedure [25]. Before the slicing procedure, the tissue undergoes warm ischemia for a variable period during the surgical procedure and its transfer to the cold oxygenated KHB buffer. During the preparation of mucosa and muscularis cores before slicing, the tissue is again exposed to (cold) ischemia. An inflammatory and subsequent fibrogenic response is provoked at the start of culture, which is unavoidable. In the context of serving as a human fibrosis (CD) model, this fibrogenesis might be a result of the ischemia since no further stimuli are needed. Of note, the majority of the genes in both the mucosa and muscularis investigated in this study were differently (downregulated) expressed in the first 24 h of incubation, and their expression recovered or increased after 48–72 h of incubation. This is thought to be the result of ischemia-reperfusion injury since very similar mRNA expression patterns of ECM-related genes have been found in another model of ischemia-reperfusion injury [26].

Interestingly, at the gene expression level, mucosa and muscularis hPCIS seem to respond quite similarly to the culture conditions. Both show increased expression of the ECM as well as ECM remodeling genes, although the MMP expression seems to be somewhat lower in muscularis hPCIS. At the protein level, there is a clear difference between the mucosa and muscularis hPCIS. The collagen secretion of mucosa hPCIS remains stable during incubation, while the secretion of collagen steadily increases in muscularis hPCIS during 72 h of incubation; Sirius Red staining also confirmed this observation.

In fibrosis, ECM not only accumulates but is also increasingly modified by collagen crosslinking enzymes [9,27]. Cross-linked collagens can only be remodeled by a few specific MMPs (MMP-1, -8, and -13) and Cathepsin K (CTSK). Interestingly, when muscularis hPCIS are stimulated with TGF-β1, the expression of *MMP-1* and *CTSK* are decreased, while the expression of *MMP-13* is increased. Together with the increased expression of *SERPINE1,* this could result in a lower collagen remodeling rate in muscularis hPCIS. Similar to ECM production, no effect on remodeling enzymes was found in mucosa hPCIS. Thus, even though the mucosa hPCIS was stimulated with TGF-β1, no pro-fibrotic effect was found. Of note, we measured the gene expression of ECM molecules and detected the pro-peptides of collagens in the culture medium rather than detecting the collagen protein itself. Previous experience taught us that the detection of collagens in precision-cut tissue slices using a western blot is unreliable. Since the pro-collagen is the product of the maturing collagen and given that this ELISA is well-established, we chose to determine pro-collagens as a measure of fibrogenesis [21,28].

MMPs might be interesting targets to treat or diagnose fibrosis [29,30]. Tissue remodeling can be determined by detecting ECM production and degradation products, i.e., so-called neo-epitopes. Since we detected both collagen 1 and 3 production and degradation products (C1M and C3M), we showed that active ECM remodeling takes place in human PCISs. Similar to the gene expression of collagens, collagen production products are lower in mucosa hPCIS. ECM degradation product levels are fairly similar between mucosa and muscularis hPCIS, while the gene expression of MMPs is generally higher in mucosa hPCIS. This could indicate that there is a difference in MMP gene translation, MMP protein secretion, and/or MMP activation in these tissue layers. Overall, no effect of incubation or treatment was found when ECM degradation products were determined. Previous studies found that in the serum of inflamed as well as stricturing (Montreal B1 and B2) CD patients, the levels of C1M and C3M were higher than in controls but similar to each other [31,32]. The differences in C1M and C3M between the hPCIS model and patients can be explained in various ways. First, C1M and C3M levels in patients are determined in serum and might result from ECM remodeling at other locations than in the intestinal tract, while in the hPCIS model, we only measured C1M and C3M produced by hPCIS [31]. Second, in the inflamed human intestine, ECM remodeling is increased, which results in increased C1M and C3M levels in the patient’s serum. We did not find differences in C1M and C3M levels at the start and end of the culture, although an increasing inflammatory (and fibrotic) signature was found. However, it is possible that the enzymatic activity was already at its maximum after 24 h of incubation and could not increase further. In future studies, it might be necessary to shorten the sampling intervals to detect an increase in MMP activity from the start of incubation to 24 h after the start of incubation. Moreover, to determine whether the detected C1M and C3M levels result from an inflammatory response in hPCIS, we could use anti-inflammatory molecules in the culture medium in future studies. As a response, lower levels of C1M and C3M would be expected.

Intestinal fibrosis most frequently develops at the transition from the ileum to the colon (ileocecal region) [2]. It might be possible that fibrosis develops in other regions as frequently as at the ileocecal region; however, due to the ‘tight’ transition at the ileocecal region, fibrosis might become symptomatic earlier [33]. We prepared hPCIS from the proximal jejunum for practical reasons. We believe that this can still be used as a translational model for intestinal fibrosis, even though intestinal fibrosis is usually located at the ileocecal region. In previous studies using the RNA-sequencing technique, PCISs prepared from the jejunum, ileum, and colon were shown to behave very similarly during incubation. It was shown that the ECM (remodeling)-related differentially expressed genes (DEGs) were very similar between the hPCIS from the jejunum and ileum during 48 h of incubation [25]. Interestingly, a similar DEG profile was found for human ileum PCISs prepared from CD ileum, indicating that healthy and diseased PCISs do not respond differently to incubation. MMP-2, MMP-3, MMP-9, and TIMP-1 release from 48-hour cultures of healthy and diseased ileum PCISs was also very similar. These authors highlighted that the lack of the underlying tissue layers, including the muscularis propria, in the hPCIS model might be the reason for the lack of discrimination between healthy and diseased PCISs [25]. Using jejunum muscularis hPCIS, we have shown that the muscularis potentially plays an important role in intestinal fibrogenesis. Further studies should demonstrate whether this holds true when ileum, colon, or CD tissue-derived muscularis hPCIS are used. Eventually, the co-culture of mucosa and muscularis hPCIS or mucosa/muscularis-specific cells could further elucidate the signaling between the different intestinal layers.

A model to study intestinal fibrosis should be stable and reliable. We found that hPCIS originating from different donors show variation in the response to culture and stimuli (e.g., TGF-β1). This is expected since the intestine is under the influence of the environment, genetics, and other (unknown) factors. To accurately mimic the variability found in human diseases, our model also incorporated this variation. To minimize the differences between tissue donors, we prepared hPCIS from the mucosa and muscularis of the same patient, thereby reducing variability and making direct comparisons between the tissues possible. Since intestines from anonymous donors were used, information regarding any cause of the variation is not available. In future studies, we will focus on personalized medicine applications and investigate the causes of the heterogeneity. A few steps have been made in the past, for example, assessing differences in metabolism between hPCIS prepared from different sexes and assessing culture-associated changes in hPCIS prepared from healthy and diseased tissues [25,34].

## 5. Conclusions

In conclusion, we showed that fibrogenesis in ex vivo cultured hPCIS is more pronounced in muscularis hPCIS. This study showed for the first time that human muscularis hPCIS can be prepared and cultured. This further improves the hPCIS model for the study of intestinal fibrosis and opens up opportunities to study the function of the muscularis propria in its native composition. Moreover, we also showed for the first time that hPCIS have active ECM remodeling and that this is different in the mucosa and muscularis. Human mucosa and muscularis PCISs could be valuable models for elucidating the mechanisms that initiate and progress intestinal fibrosis in a human ex vivo setting.

## Figures and Tables

**Table 1:** Patient characteristics.

Experiment #	Sex	Age
1	M	74
2	F	68
3	F	71
4	F	63
5	M	57
6	F	73
7	F	68
8	F	51
9	M	46
10	F	68

## Data Availability

The data underlying this article will be shared upon reasonable request to the corresponding author.

## Supplementary Materials

The following supporting information can be downloaded at: https://www.mdpi.com/article/10.3390/cells13131084/s1, Table S1: Primers; Figure S1: Viability of mucosa and muscularis hPCIS in four culture media; Figure S2: Basal gene expression of fibrosis-related genes in intestinal mucosa and muscularis; Figure S3: Presence of myofibroblasts in mucosal hPCIS. Mucosa hPCIS were incubated 24–72 h in A-DMEM-F12 with and without the addition of 5 ng/mL TGF-β1 and/or 50 ng/mL PDGF; Figure S4: Hierarchical clustering of fibrosis-related gene expression of mucosa hPCIS during incubation; Figure S5: Hierarchical clustering of fibrosis-related gene expression of muscularis hPCIS during incubation; Figure S6: Viability of mucosa and muscularis hPCIS incubated with and without TGF-β1 and/or PDGF-BB; Figure S7: Hierarchical clustering of fibrosis-related gene expression of mucosa hPCIS in the presence of TGF-β1 and/or PDGF-BB; and Figure S8: Hierarchical clustering of fibrosis-related gene expression of muscularis hPCIS in the presence of TGF-β1 and/or PDGF-BB.
