# Supplementary material for: Fibrogenesis in Human Mucosa and Muscularis Precision-Cut Intestinal Slices
Source: Cells. 2024 Jun 22;13(13):1084. doi: 10.3390/cells13131084 (PMC11240565; doi:10.3390/cells13131084)
Supplement: Supplementary file 1 [file cells-13-01084-s001.zip › cells-3048448-supplementary.pdf]

Supplementary material

Fibrogenesis in human mucosa and muscularis precision-cut intestinal slices

Supplementary table 1: Primers

Function	Gene	Forward	Reverse
ECM	*COL1A1*	GTACTGGATTGACCCCAACC	CGCCATACTCGAACTGGAAT
	COL3A1	GGAGCTGGCTACTTCTCGC	GGGAACATCCTCCTTCAACAG
	COL4A1	GGACTACCTG-GAACAAAAGGG	GCCAAGTATCTCACCTGGATCA
	COL5A1	GCCCGGATGTCGCTTACAG	AAATGCAGACGCAGGGTACAG
	COL6A1	ACACCGACTGCGCTATCAAG	CGGTCACCACAATCAGGTACTT
	FN1	CGGTGGCTGTCAGTCAAAG	AAACCTCGGCTTCCTCCATAA
	FN-ED-A	AGGACTGGCATTCAC-TGATGTG	GTCACCCTGTACCTG-GAAACTTG
Collagenases	*MMP1*	GGGGCTTT-GATGTACCCTAGC	TGTCACACGCTTTTGGGGTTT
	MMP8	TTTTGATGCCGAA-GAAACATGGA	GTGAGCGAGCCCCAAAGAA
	MMP13	CCAGACTTCACGATGG-CATTG	GGCATCTCCTCCATAATTTGGC
	CTSK	GCAGAAGAACCGGGG-TATTGA	GAAGGAGGTCAGGCTTGCAT
Gelatinases	*MMP2*	TACAGGATCATTGGC-TACACACC	GGTCACATCGCTCCAGACT
	MMP9	AGACCTGGGCAGAT-TCCAAAC	CGGCAAGTCTTCCGAGTAGT
Stromelysin	*MMP3*	ACAAAGGATA-CAACAGGGACCAA	ACCGAGTCAGGTCTGTGAGT
Macrophage elastase	*MMP12*	GGAATCCTAGCCCATGCTTTT	CATTACGGCCTTTGGATCACT
Protease inhibitors	*TIMP1*	AATTCCGACCTCGTCATCAG	TGCAGTTTTCCAGCAATGAG
	TIMP2	GCTGCGAGTGCAAGATCAC	TGGTGCCCGTTGATGTTCTTC
	SERPINE1	ACCGCAACGTGGTTTTCTCA	TTGAATCCCATAGCTGCTT-GAAT
Pro-collagen processing	*ADAMTS2*	GTGCATGTGGTGTATCGCC	AGGACCTCGATGTTGTAGTCA
	ADAMTS14	CAGCACAGACTTTCGGGGAC	GGAGTGTCGTGGTAGTGTGG
Collagen crosslinking	*LOX*	GCCGACCAAGA-TATTCCTGGG	GCAGGTCATAGTGGCTAAACTC
Differentiation markers	*DES*	GAGACCATCGCGGCTAA-GAAC	GTGTAGGACTGGATCTGGTGT
	ACTA2	AAAAGACAGCTAC-GTGGGTGA	GCCATGTTCTATCGGGTACTTC
	VIM	AGTCCACTGAGTACCGGA-GAC	CATTTCACGCATCTGGCGTTC
	ROCK2	AACGTCAGGATGCAGATGGG	CAGCCAAAGAGTCCCGTTCA

	FAP	TGAACGAGTATGTTTGCAG-TGG	GGTCTTTGGACAATCCCATGT
Proliferation	*CCND1*	GCTGCGAAGTGGAAACCATC	CCTCCTTCTGCACACATTTGAA
	MKI67	AC-GCCTGGTTACTATCAAAAGG	CAGACCCATTTACTTGTGTT-GGA
Reference Gene	*YWHAZ*	CCTGCATGAAGTCTG-TAACTGAG	GACCTACGGGCTCCTACAACA

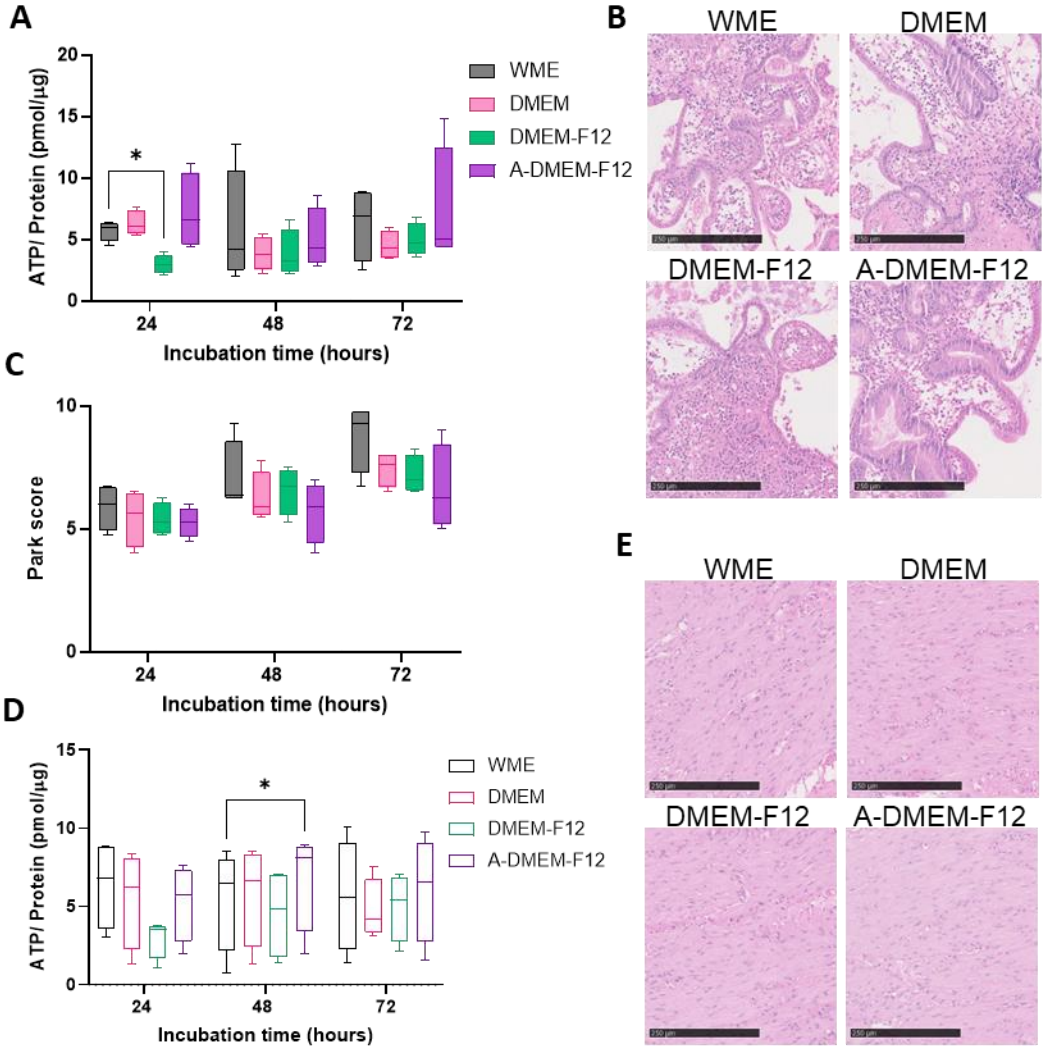

Supplementary figure S1: Viability of mucosa and muscularis hPCIS in four culture media. Mu-cosa and muscularis hPCIS were incubated in WME, DMEM, DMEM-F12 or Advanced(A)-DMEM-F12 for 24-72 hours. After incubation ATP/protein ratio of **(a)** mucosa and (**d**) muscularis was determined; Representative images of **(b)** mucosa hPCIS and (**e**) muscularis hPCIS Bars in-dicate 250 µm (10x magnification); (**c**) Mucosa HE staining were scored using an adapted Park scoring system (Bigaeva et al 2019), a higher score indicates lower viability of the tissue (maxi-mum score is 10).

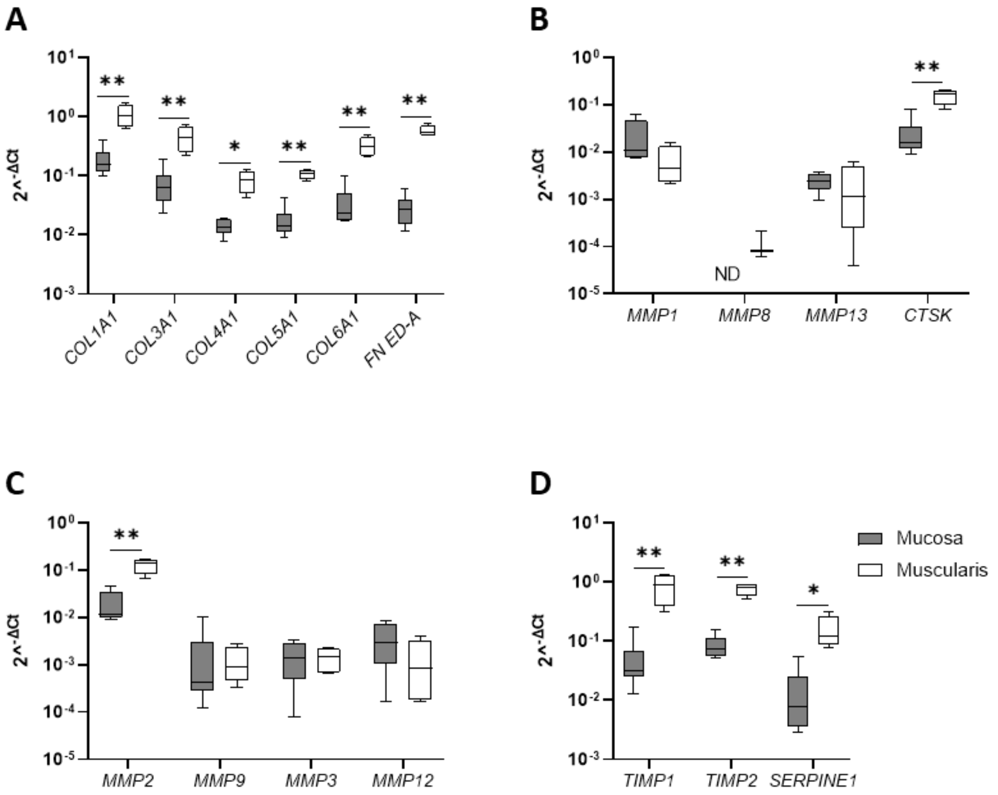

Supplementary figure S2: Basal gene expression of fibrosis related genes in intestinal mucosa and muscularis. Mucosa and muscularis hPCIS were collected after the slicing procedure. Gene expres-sion analysis of: **(a)** ECM related genes; **(b)** collagenases; **(c)** MMPs and; **(d)** inhibitors of MMPs was performed. *p<0.05, **p<0.01 compared to mucosa. Bars indicate minimum, maximum and median values. Closed bars indicate values obtained from mucosa hPCIS, open bars show muscu-laris.

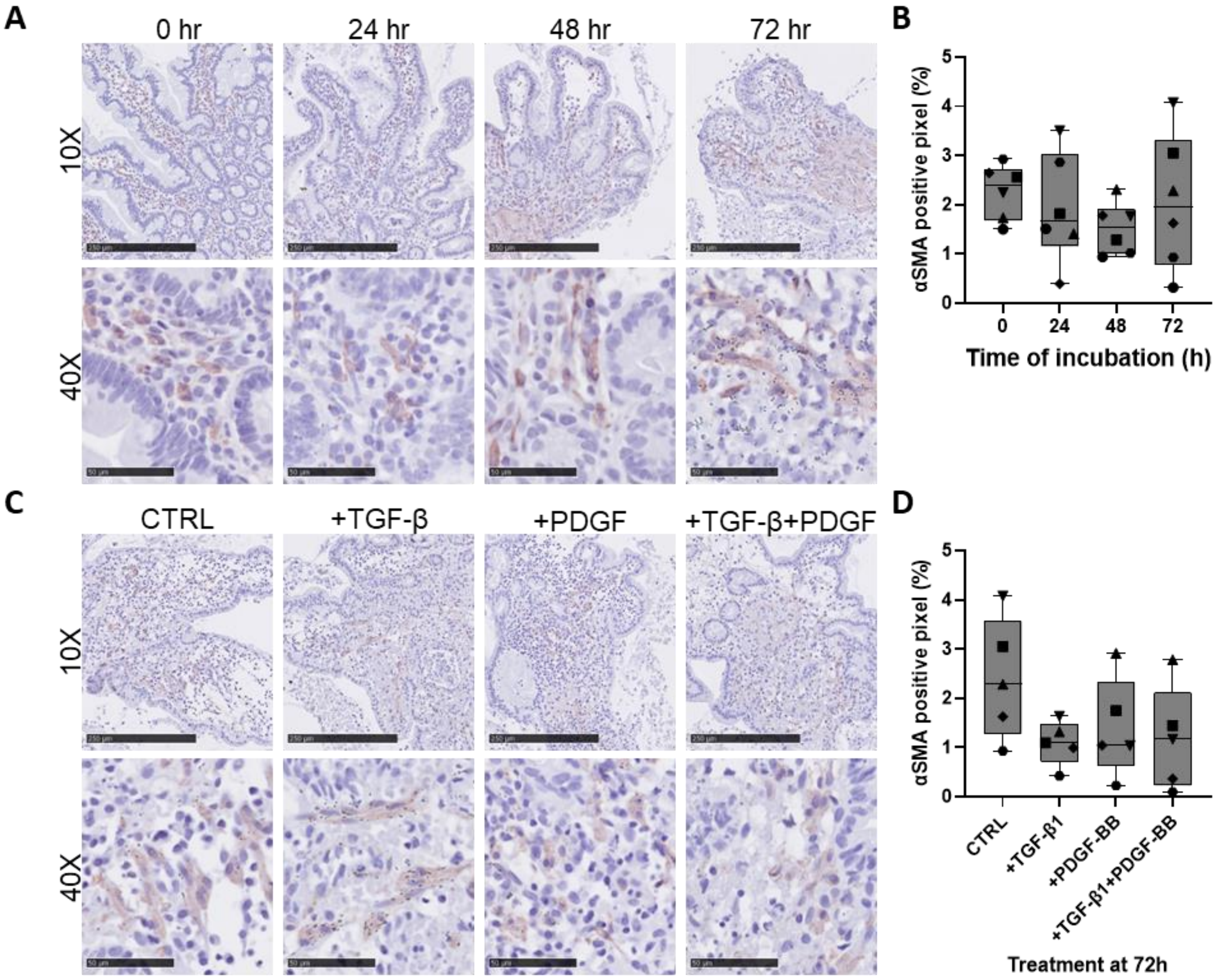

Supplementary figure S3: Presence of myofibroblasts in mucosal hPCIS. Mucosa hPCIS were incubated 24-72 hours in A-DMEM-F12 with and without the addition of 5ng/mL TGF-β1 and/or 50ng/mL PDGF. Directly after slicing and after incubation mucosa hPCIS were fixated and subsequently IHC for α-SMA was performed. Representative images of; **(a)** mucosa hPCIS during incubation and; **(c)** after 72 h of incubation with and without TGF-β1 and/or PDGF-BB and αSMA positive pixel count (ns) (**b** (n=6), **d** (n=5)). Bars indicate 250 μm (10x magnification) and 50 μm (40x magnification).

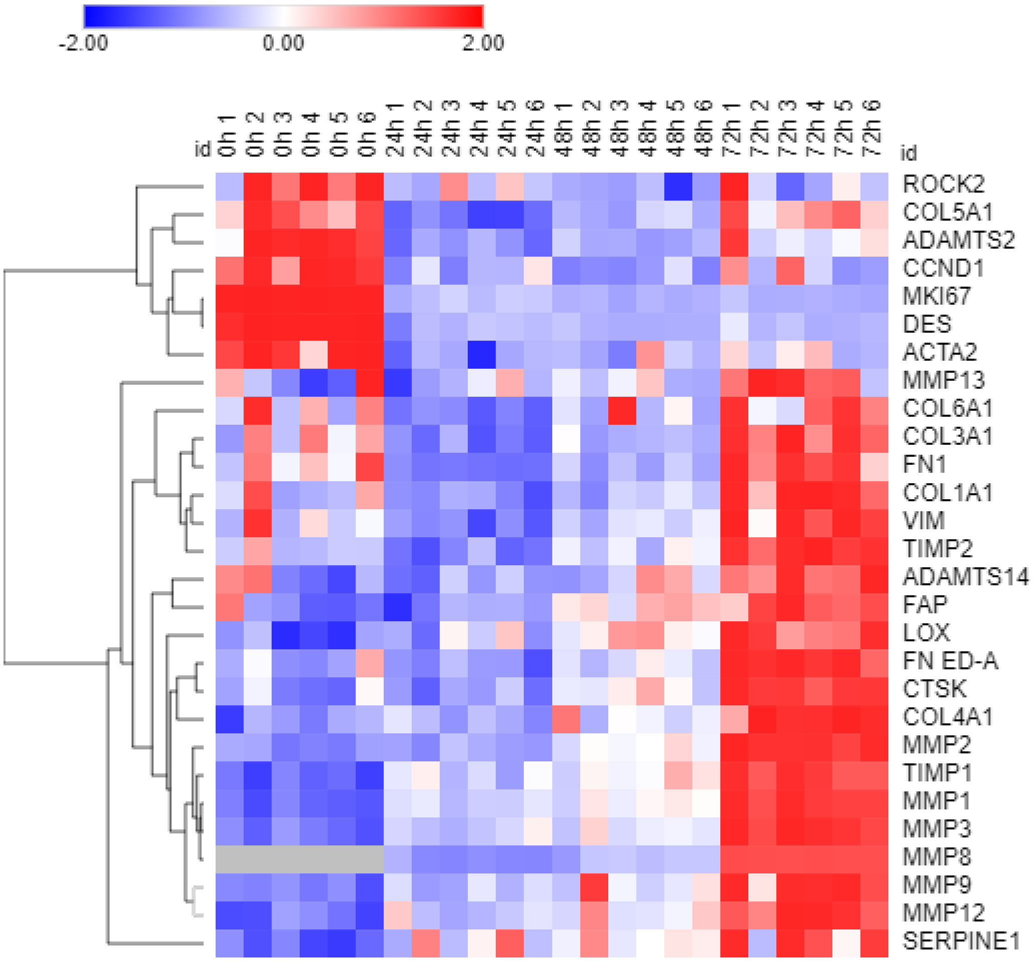

Supplementary figure S4: Hierarchical clustering of fibrosis related gene expression of mucosa hPCIS during incubation. Mucosa hPCIS were incubated 24-72 hours in A-DMEM-F12. Subsequently RT-qPCR analysis of fibrosis-related genes was performed. Z-values were calculated using the 2-ΔCt-average and 2-ΔCt-standard deviation from individual experiments. Heatmaps were generated using Morphous online software (https:// software.broadinstitute.org/morpheus/). Hierarchical clustering analysis was performed by average-linkage clustering method using Pearson correlation.

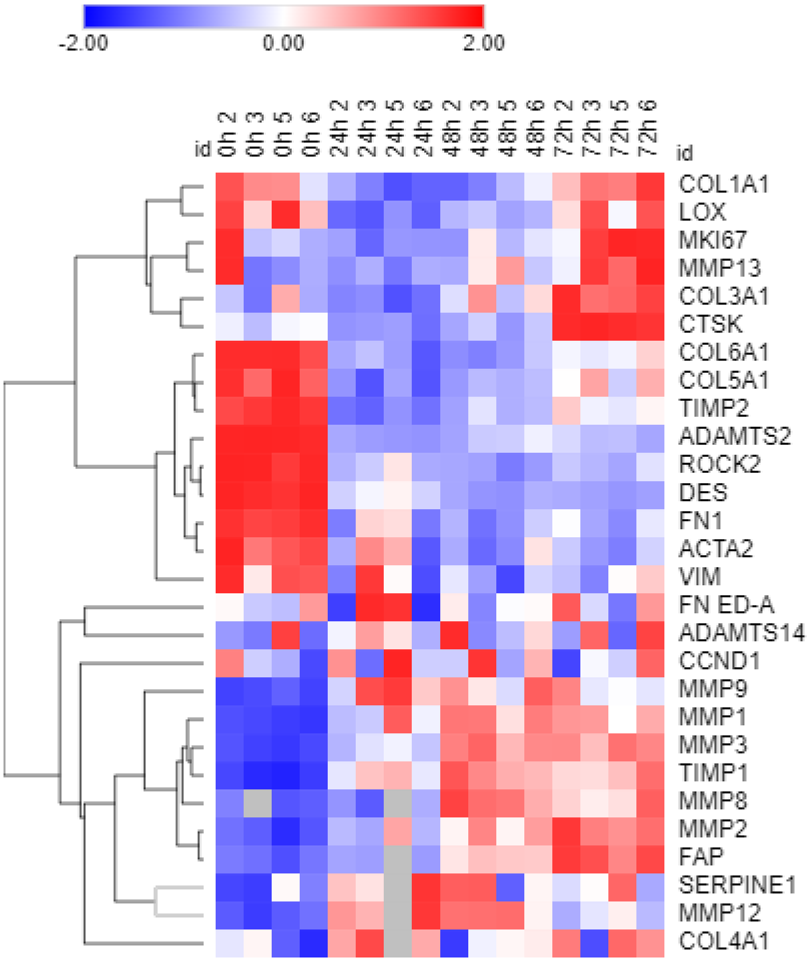

Supplementary figure S5: Hierarchical clustering of fibrosis related gene expression of muscularis hPCIS during incubation. Muscularis hPCIS were incubated 24-72 hours in A-DMEM-F12. Subse-quently, RT-qPCR analysis of fibrosis-related genes was performed. Z-values were calculated us-ing the 2-ΔCt-average and 2-ΔCt-standard deviation from individual experiments. Heatmaps were generated using Morphous online software (https://software.broadinstitute.org/morpheus/). Hi-erarchical clustering analysis was performed by average-linkage clustering method using Pearson correlation.

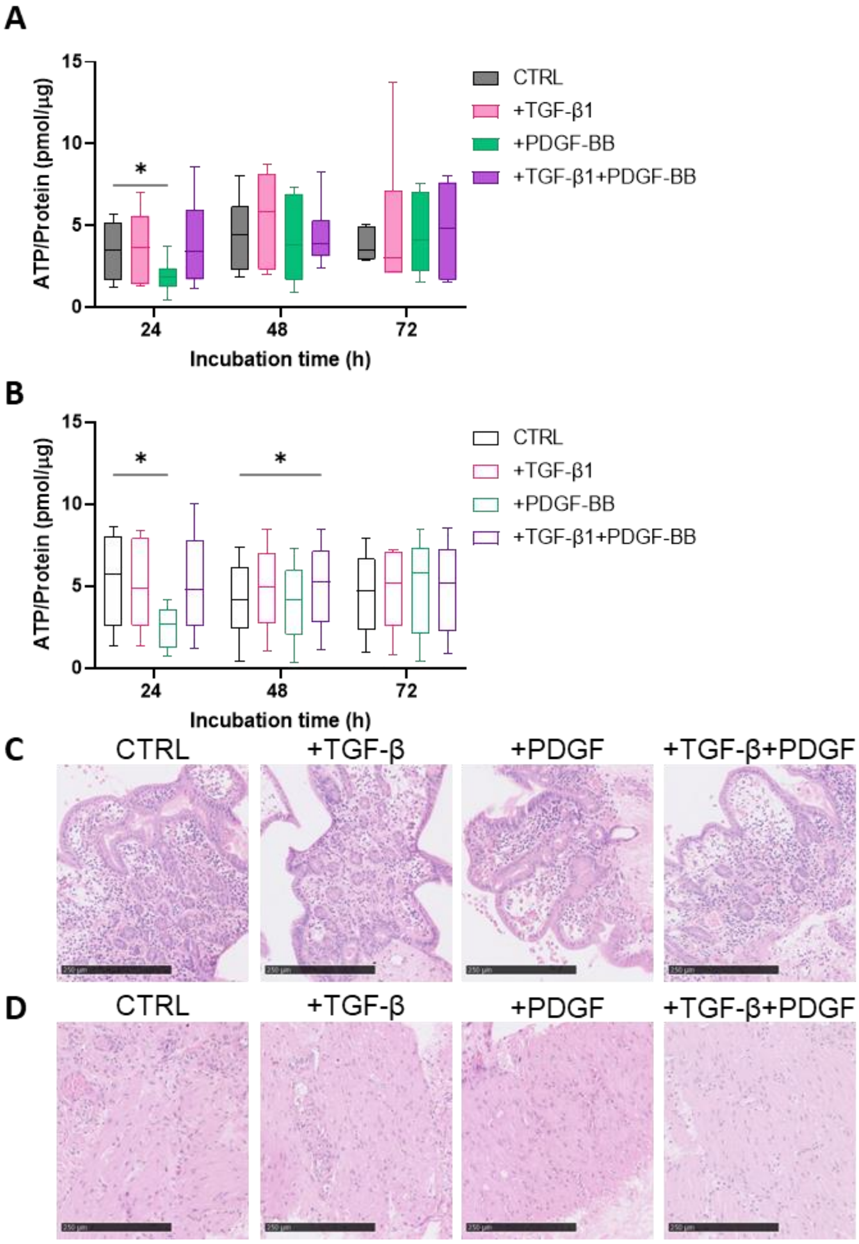

Supplementary figure S6: Viability of mucosa and muscularis hPCIS incubated with and without TGF-β1 and/or PDGF-BB. Mucosa and muscularis hPCIS were incubated 24-72 hours in A-DMEM-F12 with and without the addition of 5ng/mL TGF-β1 and/or 50ng/mL PDGF. After incubation, ATP/protein ratio of **(a)** mucosa and; **(b)** muscularis was determined. Representative images of **(c)** mucosa hPCIS and **(d)** muscularis hPCIS. Bars indicate 250 µm (10x magnification).

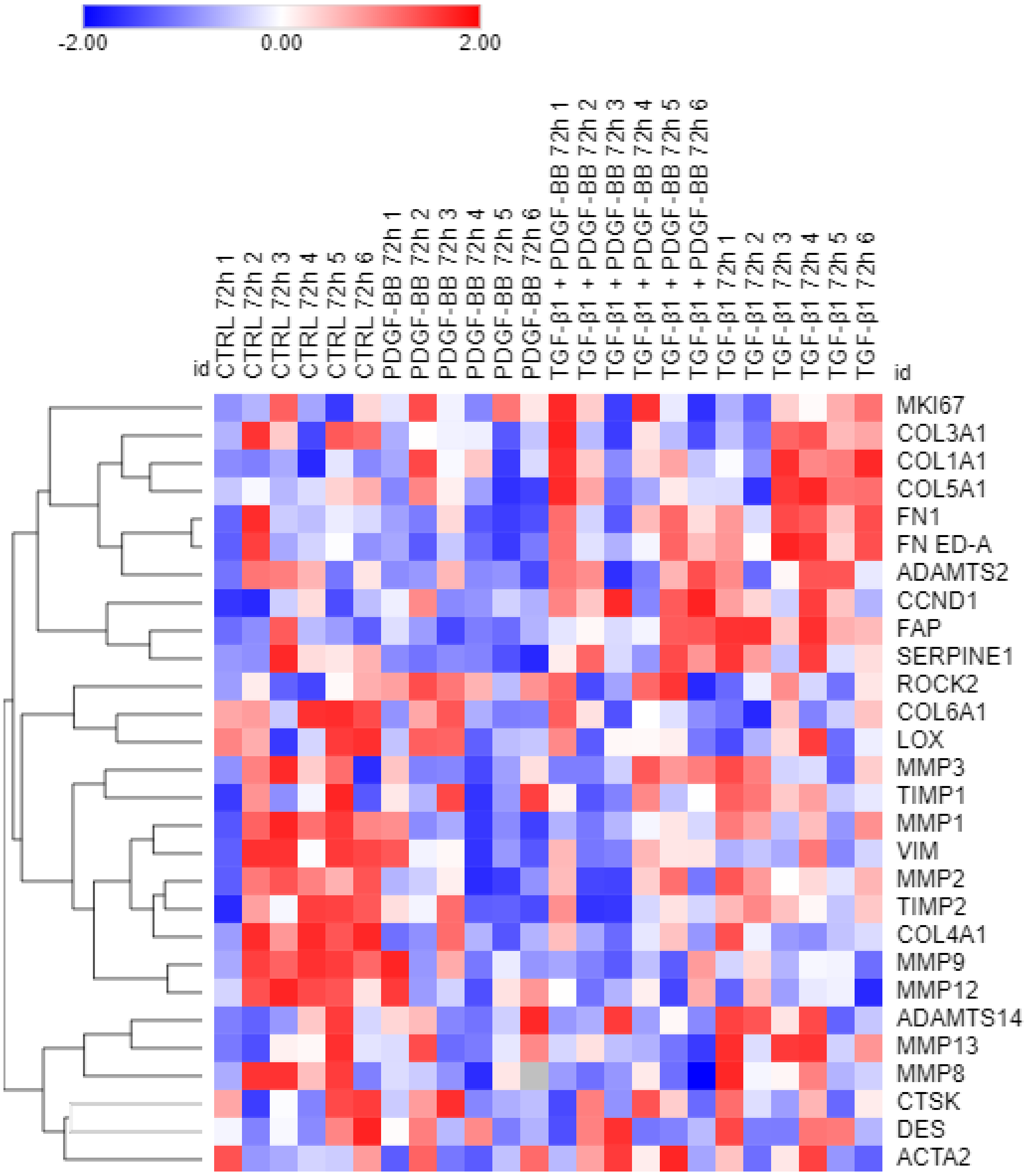

Supplementary figure S7: Hierarchical clustering of fibrosis related gene expression of mucosa hPCIS in presence of TGF-β1 and/or PDGF-BB. Mucosa hPCIS were incubated for 72 hours with or without TGF-β1 and/or PDGF-BB. Subsequently, RT-qPCR analysis of fibrosis-related genes was performed. Z-values were calculated using the 2-ΔCt-average and 2-ΔCt-standard deviation from individual experiments. Heatmaps were generated using Morphous online software (https:// software.broadinstitute.org/morpheus/). Hierarchical clustering analysis was performed by average-linkage clustering method using Pearson correlation.

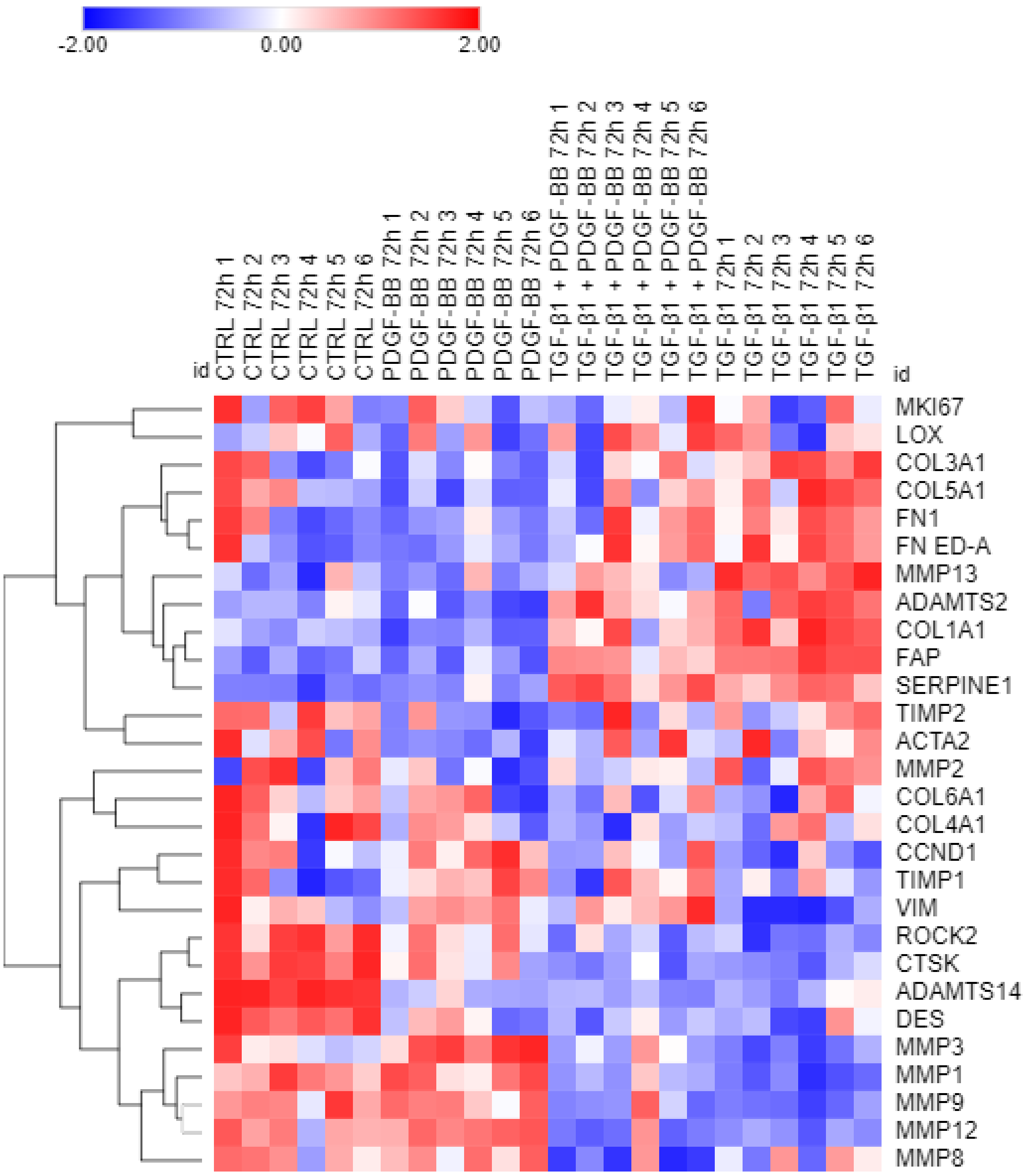

Supplementary figure S8: Hierarchical clustering of fibrosis related gene expression of muscularis hPCIS in presence of TGF-β1 and/or PDGF-BB. Muscularis hPCIS were incubated for 72 hours with or without TGF-β1 and/or PDGF-BB. Subsequently, RT-qPCR analysis of fibrosis-related genes was performed. Z-values were calculated using the 2-ΔCt-average and 2-ΔCt-standard deviation from individual experiments. Heatmaps were generated using Morphous online software (https:// software.broadinstitute.org/morpheus/). Hierarchical clustering analysis was performed by average-linkage clustering method using Pearson correlation.
